# No Influence of Emotional Faces or Autistic Traits on Gaze-Cueing in General Population

**DOI:** 10.3389/fpsyg.2022.864116

**Published:** 2022-04-26

**Authors:** Shota Uono, Yuka Egashira, Sayuri Hayashi, Miki Takada, Masatoshi Ukezono, Takashi Okada

**Affiliations:** Department of Developmental Disorders, National Institute of Mental Health, National Center of Neurology and Psychiatry, Kodaira, Japan

**Keywords:** attention orienting, autistic traits, facial expressions, gaze direction, gaze-cueing effect

## Abstract

The present study addressed the controversial issue of whether autistic traits in the general population are associated with the automatic and fundamental aspects of joint attention through eye gaze. Specifically, we examined whether the degree of autistic traits is associated with the magnitude of reflexive attention orienting in the direction of another’s eye gaze embedded in neutral and emotional (angry, fearful, and happy) faces. The cue stimuli changed gaze direction and facial expressions simultaneously. Participants were asked to detect a target that appeared at the left or right of the cue stimuli. The results revealed a robust gaze-cueing effect, such that the reaction time to the target was shorter under the gazed-at-target condition than under the non-gazed-at-target condition. However, emotional expressions did not modulate the gaze-cueing effect. Furthermore, individual differences in autistic traits and emotional characteristics (social anxiety, alexithymia, and emotional disturbances) did not influence the magnitude of the gaze-cueing effect. Although the ability to orient attention in the direction of another’s gaze is a fundamental function of social development, the gaze-cueing effect measured in a controlled experiment might not be an elaborate representation of the current social cognitive function, at least in typically developing adults.

## Introduction

Humans have eyes with distinctive features that partially reveal a person’s thinking to others (i.e., a white sclera and dark iris; Kobayashi and Kohshima, 2001). Eye gaze tells us what a person is attending to and helps us know about their mental state (Baron-Cohen, 1995). Gaze perception and joint attention appear in early development; they have crucial roles in the development of social cognition, such as empathy and affective evaluation (Stephenson et al., 2021). However, there are large individual differences in these functions. For instance, individuals with autism-spectrum disorder (ASD), characterised by difficulty in social communication (American Psychiatric Association, 2013), show less attention to (e.g., Pelphrey et al., 2002) and reduced understanding of eye gaze (e.g., Baron-Cohen et al., 2001a). A deficit in joint attention, in which two people attend to the same object or event, is an early marker of emerging autism (Mundy et al., 1994).

The autistic phenotypes are continuously distributed across the general population (Constantino and Todd, 2003). Consistent with this distribution, previous studies have demonstrated that individuals with ASD and individuals with high-autistic traits show similar difficulty in social cognition, such as attention orienting in the direction of an eye gaze (e.g., Zhao et al., 2015) and detecting emotional facial expressions (e.g., Sato et al., 2017). Thus, investigations of how autistic traits affect social cognitive function provide important contributions to the understanding of developmental variability in the general population. The present study addressed the controversial issue of whether autistic traits in the general population are associated with gaze-triggered attention, which is thought to be important for joint attention. Specifically, we examined whether the degree of autistic traits is associated with the magnitude of reflexive attention orienting in the direction of another’s eye gaze embedded in neutral and emotional faces.

Many experimental studies have demonstrated that humans orient their attention in the direction of another’s gaze (see Frischen et al., 2007 for review). A pioneering study examined the attentional effect of the gaze cue that was nonpredictive of the location of a subsequently presented target (Friesen and Kingstone, 1998). Participants detected, localised or discriminated the target stimulus that appeared either to the left or right of the cue stimulus. The reaction time (RT) to the target under the gazed-at-target (i.e., congruent) condition was shorter than the RT to the target under the non-gazed-at-target (i.e., incongruent) condition, irrespective of the task demands. The gaze-cueing effect (i.e., the difference in RT between incongruent and congruent conditions) is evident even when the target is more likely to appear at the non-cued location than at the cued location (e.g., Driver et al., 1999) and the cue is presented subliminally to the observers (e.g., Sato et al., 2007). These findings indicate that gaze cues automatically trigger the observer’s attentional orienting. Previous studies have demonstrated that gaze cues reflexively orient attention for toddlers (e.g., Chawarska et al., 2003), young children (e.g., Kylliäinen and Hietanen, 2004) and adults with ASD (e.g., Pruett Jr. et al., 2011; however, see Ristic et al., 2005). Although clinical observations revealed deficits in joint attention under actual interactions (e.g., Okada et al., 2003), these findings suggest that individuals with ASD show an intact gaze-cueing effect under experimentally controlled situations.

It is reasonable to presume that the gaze-cueing effect of emotional faces can allow differentiation between individuals with and without autism, considering the social importance of facial expressions that communicate a positive or negative feeling of an expresser and the valence of an object (Blair, 2003). Although previous studies in typically developing individuals have reported mixed findings when static facial expressions were the cue stimuli, there is increasing evidence that dynamic facial expressions, changing from neutral to emotional expressions, can modulate the magnitude of the gaze-cueing effect (see Dalmaso et al., 2020 for review). In particular, dynamic fearful faces induce an enhanced gaze-cueing effect compared with neutral faces (Tipples, 2006; Uono et al., 2009a; Graham et al., 2010; Lassalle and Itier, 2013, 2015b; Neath et al., 2013; Dawel et al., 2015; McCrackin and Itier, 2018; Chow et al., 2021; but see Bayless et al., 2011) and other emotional faces (Putman et al., 2006; Bayless et al., 2011; Neath et al., 2013; McCrackin and Itier, 2018, 2019; Chow et al., 2021; however, see Fichtenholtz et al., 2007, 2009; Graham et al., 2010; Lachat et al., 2012b; Uono et al., 2009a; Lassalle and Itier, 2015a,b). Evidence suggests that angry (Lassalle and Itier, 2013, 2015b; Pecchinenda and Petrucci, 2016; Liu et al., 2019) and happy faces (Graham et al., 2010; McCrackin and Itier, 2018, 2019) can enhance the gaze-cueing effect compared to neutral faces, although the enhancement could be less than with fearful faces (Uono et al., 2009a; McCrackin and Itier, 2018, 2019). With respect to individuals with ASD, only two studies have investigated the influence of facial expressions on the gaze-cueing effect (de Jong et al., 2008; Uono et al., 2009b). These studies demonstrated that dynamic fearful expressions enhance the gaze-cueing effect compared with neutral faces in typically developing individuals, but not in individuals with ASD. However, these findings are not necessarily conclusive with respect to whether the gaze-cueing effect in individuals with ASD is not facilitated in the fearful face condition or is enhanced in the neutral face condition because there were no group differences in the gaze-cueing effect under either condition.

Consistent with investigations of clinically diagnosed ASD, previous studies have reported that the degree of autistic traits in the general population influences the gaze-cueing effect of emotional and neutral faces. Previous studies using neutral faces as cues have reported that, compared to individuals with high-autistic traits, individuals with low autistic traits show an enhanced gaze-cueing effect (Bayliss et al., 2005) when a specific kind of target appears (integrated vs. scrambled objects: Bayliss and Tipper, 2005; voice vs. tone: Zhao et al., 2015). However, other studies did not find such an association of autistic traits with the gaze-cueing effect of neutral faces (Lachat et al., 2012a; de Araujo et al., 2021). Lassalle and Itier (2015a) used dynamic cues which changed emotional expression after gaze deviation and showed that the gaze-cueing effect of fearful faces was larger than that of happy faces in participants with high-autistic traits. Further, the smaller gaze-cueing effect of happy faces was associated with higher autistic traits. Recent studies with large sample sizes have reported contradictory findings concerning the relationship between autistic traits and the gaze-cueing effect of emotional faces. Similar to Lassalle and Itier (2015a), McCrackin and Itier (2019) used the dynamic cues; they found that a stronger autistic trait (i.e., attention to detail) was associated with a weaker gaze-cueing effect from fearful and happy faces. In contrast, Talipski et al. (2020) used static emotional faces which appeared prior to the change of their gaze direction; they did not find any emotional enhancement of the gaze-cueing effect, nor did they find any association between autistic traits and the gaze-cueing effect of emotional faces. Thus, the evidence is not conclusive regarding the association between the gaze-cueing effect and autistic traits in the general population.

In the present study, we first aimed to replicate the previous studies that demonstrated emotional enhancement of the gaze-cueing effect. We employed dynamic gaze cues that demonstrated enhancement of the gaze-cueing effect for dynamic fearful faces versus neutral faces in previous studies (Uono et al., 2009a,b). Neutral expression and direct gaze began to change simultaneously to an emotional expression and an averted gaze under an emotional face condition, while only the gaze direction changed from direct to averted under the neutral face condition. The present study included fearful faces as well as angry and happy faces as the cue stimuli because there is evidence that other emotional faces enhance the gaze-cueing effect compared to neutral faces (angry: Lassalle and Itier, 2013, 2015b; Pecchinenda and Petrucci, 2016; Liu et al., 2019; happy: Graham et al., 2010; McCrackin and Itier, 2018, 2019), although the enhancement could be less than with fearful faces (Uono et al., 2009a; McCrackin and Itier, 2018, 2019). Our primary objective was to examine whether the degree of autistic traits modulates the gaze-cueing effect of emotional and neutral faces in the general population. In addition to the Autism-Spectrum Quotient (AQ; Baron-Cohen et al., 2001b), we used the Social Responsiveness Scale, second edition (SRS-2), which is a specific measure of social impairment associated with ASD (Constantino and Gruber, 2012). The convergence of the results between the AQ and SRS could strengthen the validity of the evidence. Based on the previous studies in clinical settings (de Jong et al., 2008; Uono et al., 2009b) and the general population (McCrackin and Itier, 2019) using dynamic emotional faces as the cue stimuli (though gaze direction changed prior to facial expressions in McCrackin and Itier, 2019), we hypothesised that individuals with higher AQ would exhibit a smaller gaze-cueing effect, particularly for emotional faces. Additionally, we measured potential confounding factors (social anxiety, alexithymia and mood disturbances) using self-report questionnaires. Previous studies have indicated that these three factors contribute to the difficulty in processing facial expressions (e.g., Machado-de-Sousa et al., 2010; Grynberg et al., 2012; Krause et al., 2021) and they are related to autistic traits (e.g., Rosbrook and Whittingham, 2010; Freeth et al., 2013; Foulkes et al., 2015). People with autism have higher rates of these problems compared to the general population (e.g., Spain et al., 2018; Kinnaird et al., 2019; Hossain et al., 2020). To investigate the association between autistic traits such as social difficulty and detail-focused processing style, which are common in people with high-autistic traits and the gaze-cueing effect, we planned to control the level of each emotional trait. Furthermore, to confirm whether the explicit processing of emotion contributes to the gaze-cueing effect of emotional faces, the ability to recognise emotions was measured using the label-matching paradigm (Uono et al., 2017).

## Materials and Methods

### Participants

We list demographic data of the participants in Table 1. Seventy-nine Japanese adults were recruited through advertisement on an institutional website. The sample size was determined to detect a medium effect size for the planned correlation analysis between autistic traits and task performance (*r* = 0.33; Lassalle and Itier, 2015a). For a statistical power of 0.80 and an alpha of 0.05, a sample size of 69 was estimated to be sufficient to detect such an effect (G*Power software; Faul et al., 2007). Intellectual ability was evaluated using the Japanese version of the Wechsler Adult Intelligence Scale, fourth edition (Ueno et al., 2018). The intelligence quotients of all participants were within the normal range. All participants had normal or corrected-to-normal visual acuity.

**Table 1:** Participants’ demographic data (*N* = 79).

Sex	Men:women	
	21:58	
	Mean (*SD*)	Range
Age	25.00 (10.09)	18–55
*Autistic traits*		
AQ	18.10 (7.62)	4–36
AQ, four-point scale	108.46 (16.60)	58–148
SRS-2	46.24 (23.60)	3–107
*Traits frequently comorbid of autism*		
LSAS	51.75 (23.47)	3–106
TAS-20	45.01 (11.71)	23–74
POMS 2	48.16 (9.71)	30–80
*Intellectual ability*		
FSIQ	109.20 (9.69)	86–136

AQ: autism-spectrum quotient; FSIQ: full-scale intelligence quotient; LSAS: Liebowitz social anxiety scale; POMS 2: profile of mood states, second edition; SD: standard deviation; SRS-2; social responsiveness scale, second edition; and TAS-20: Toronto alexithymia scale.

This study is a part of a large research project: The Elucidation of Pathology in Neurodevelopmental Disorders Based on the Understanding of Neuropsychological and Neurophysiological Function (A2021-125). This project’s protocol was approved by the Ethics Committee of the National Centre of Neurology and Psychiatry, Japan; the study was performed in accordance with the Ethical Guidelines for Medical and Health Research Involving Human Subjects. All participants provided written informed consent before they participated in the study.

### Questionnaires: Autistic Traits

We assessed participants’ autistic traits using self-report questionnaires. We did not exclude participants from analysis based on a clinical cut-off score in each questionnaire because we aimed to investigate individual differences in the gaze-cueing effect of emotional facial expressions and its underlying mechanisms.

#### Autism Spectrum Quotient

Autistic traits were measured using the Japanese version (Wakabayashi et al., 2006) of the AQ (Baron-Cohen et al., 2001b). The AQ includes 50 items that evaluate five domains relevant to autistic symptoms (social skill, communication, attention-switching, attention to detail and imagination). Participants were asked to select ‘agree’, ‘slightly agree’, ‘slightly disagree’ or ‘disagree’ for each item. The possible range of the original scoring method was 0–50. The mean of the overall AQ scores in the present study was slightly lower than the mean from a large sample study in Japan (Wakabayashi et al., 2006; mean ± *SD* = 20.7 ± 6.38). The scores of four participants were above the cut-off in the Japanese version of the AQ (≥33: Wakabayashi et al., 2006). To emphasise individual differences in autistic traits, we used the total score of the 4-point scale for each item (possible range: 50–200; Austin, 2005), which was also used in McCrackin and Itier (2019) to demonstrate the association between autistic traits and the gaze-cueing effect.

#### Social Responsiveness Scale, Second Edition

The deficits in social behaviour associated with ASD (social awareness, social cognition, social communication, social motivation and restricted interests and repetitive behaviour) were measured by the Japanese version of the social responsiveness scale, second edition (SRS-2; Constantino and Gruber, 2012), which is currently undergoing standardisation. The scale includes 65 items using a 4 point Likert scale (1 = ‘not true’; 4 = ‘almost always true’). Here, we have reported the totalled score for several domains. The scores of the SRS-2 were highly associated with the scores of the AQ in the present study (*r* = 0.720, *p* < 0.001).

### Questionnaires: Characteristics Relevant to Autism

To assess the levels of social anxiety, alexithymia, and mood disturbances, we used the Liebowitz Social Anxiety Scale (LSAS; Liebowitz, 1987), Toronto Alexithymia Scale (TAS-20; Bagby et al., 1994) and Profile of Mood States, second edition (POMS 2; Heuchert and McNair, 2012), respectively. Preliminary analysis confirmed positive correlations between autistic traits measured by AQ and social anxiety (*r* = 0.617, *p* < 0.001), alexithymia (*r* = 0.550, *p* < 0.001) and mood disturbances (*r* = 0.547, *p* < 0.001).

#### Liebowitz Social Anxiety Scale

The degree of social anxiety was measured using the Japanese version (Asakura et al., 2002) of the LSAS (Liebowitz, 1987). Participants rated the degree to which they feel anxiety or fear under 11 social interaction and 13 performance situations (0 = ‘not at all’; 3 = ‘very strongly’) and how often they avoid such situations (0 = ‘not at all’; 3 = ‘more than two-thirds’) using a 4-point Likert scale. In the present study, the total score (possible range = 0–144), which summed the anxiety and avoidance component scores, was similar to the total score from a large sample study in Japan (Kitazoe et al., 2014; mean ± *SD* = 50.3 ± 23.5). Previous studies have demonstrated the association between autistic traits and social anxiety measured by the LSAS (Freeth et al., 2013; Kitazoe et al., 2014).

#### Toronto Alexithymia Scale (TAS-20)

The degree of alexithymia was assessed by the Japanese version (Komaki et al., 2003) of the TAS-20 (Bagby et al., 1994). The questionnaire consists of 20 items concerning difficulties of identifying and describing feelings, as well as difficulties of externally oriented thinking. Participants responded to each item using a 5-point Likert scale (1 = ‘strongly disagree’; 5 = ‘strongly agree’). The total score was calculated by adding the scores of the three domains (possible range = 20–100). The mean of the total scores was slightly lower than the mean of healthy Japanese individuals in a large sample study (Moriguchi et al., 2007; mean ± *SD* = 48.3 ± 8.9). The scores of 10 participants were above the cut-off on the Japanese version of the TAS-20 (≥59). Previous studies have demonstrated the association between autistic traits and alexithymia measured by the TAS-20 (Foulkes et al., 2015; Yamawaki and Kono, 2020).

#### Profile of Mood States, Second Edition

Participants’ mood states in the previous 7 days were measured using the Japanese version (Yokoyama and Watanabe, 2015) of the POMS 2 (Heuchert and McNair, 2012). The scale consists of 35 items. Participants indicated the strengths of various mood states that they experienced (anger–hostility, confusion–bewilderment, depression–dejection, fatigue–inertia, tension–anxiety, vigour–activity and friendliness) using a 5-point Likert scale (0 = ‘not at all’; 4 = ‘very much’). The total mood disturbance score was calculated by adding all subscale scores except for the vigour activity and friendliness score, then subtracting the vigour–activity score. The total score (possible range = –20–100) was transformed into a standardised score based on the participant’s sex (possible range = 30–90); its mean was within the normal range of mood states (40–59). The scores of 10 participants were above the cut-off on the Japanese version of the POMS 2 (≥60). A previous study in Japan reported that mood disturbance measured by the previous version of the POMS is associated with autistic traits (Ise et al., 2015).

### Stimuli

**Figure 1:**
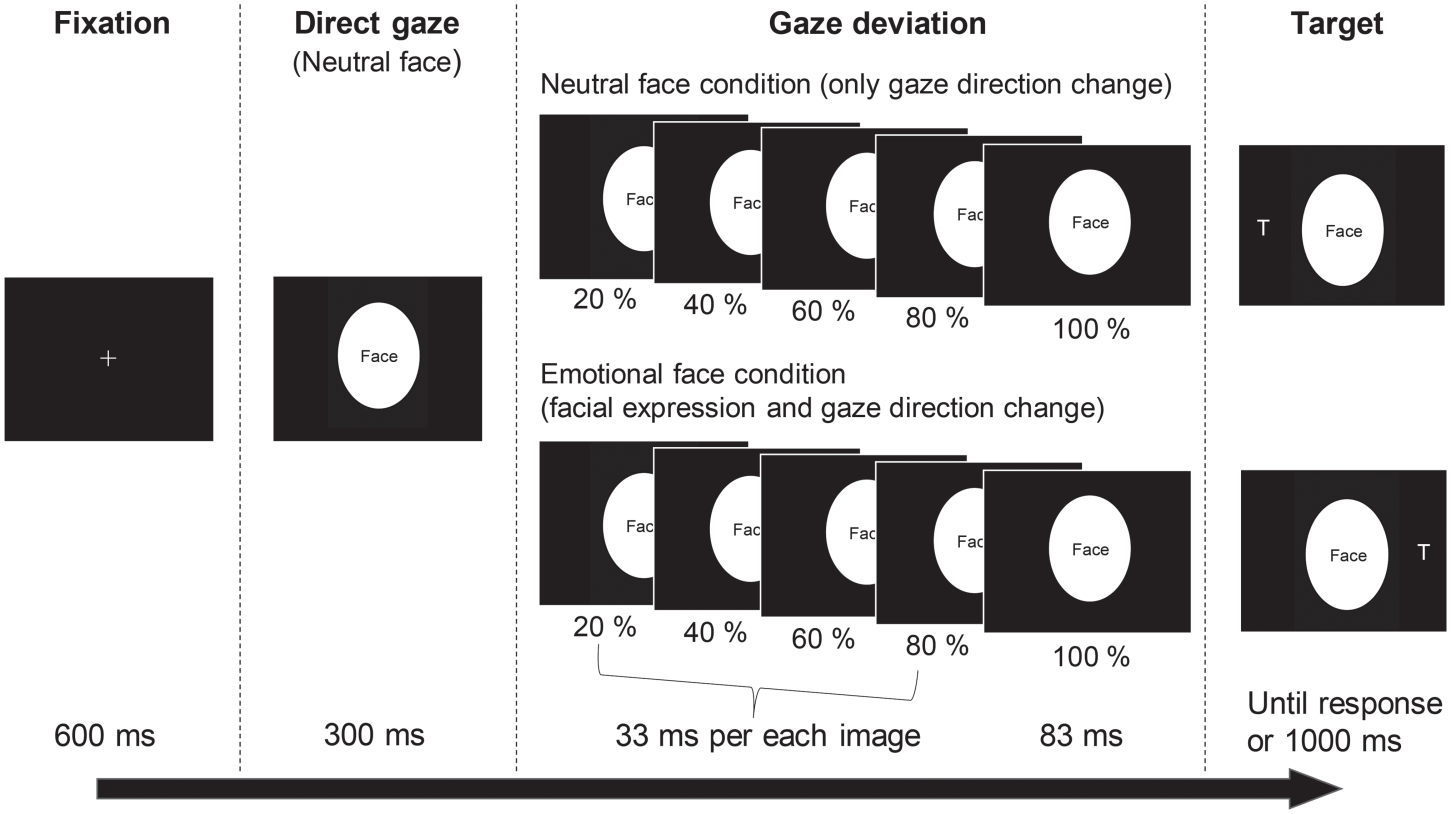
The trial sequence of the gaze-cueing task. The cue stimuli were replaced with ‘Face’ because of a copyright restriction (see Uono et al., 2009a,b for the real facial images).

We selected photographs of two models (one male [JJ] and one female [MO]) with neutral and emotional (angry, fearful and happy) faces (Ekman and Friesen, 1976). Four intermediate images between the neutral (0%) and each emotional expression (100%) were created in 20% steps using computer morphing software (FUTON, ATR-Promotions). The gaze direction was manipulated using Photoshop software (Adobe). The irises and pupils of the eyes were cut from the original photographs and pasted to fit the right or left position of the eyes; the positions matched each percentage of the intermediate photographs. The photographs were cropped in an ellipse 4.2° wide and 5.7° high to exclude the hair and background. The stimuli were sequentially presented from 0% (neutral) to 100% (original extent of emotion) under the angry, fearful and happy face conditions (Figure 1). The first (0%) image was presented for 300 ms. Then, each intermediate image (20%, 40%, 60% and 80%) was presented for 33 ms. The last 100% image remained on the screen until the trial was ended by the participant’s response or a time limit (1,000 ms). For the neutral face condition, only the gaze direction was gradually changed to the left or right. In total, 82 photographs were used as cue stimuli, consisting of emotion (neutral, angry, fearful, and happy) × gaze direction (four intermediate positions and an end position each for right and left) × person (two models), plus a neutral face with direct gaze for each model. The target, a letter T (0.5° wide and 0.8° high), was presented 8.4° to the left or right of the cue stimuli.

### Apparatus

Stimulus presentation and data acquisition were controlled by Presentation software (version 22.1; Neurobehavioral Systems; Berkeley, CA, United States) on a Windows computer (XPS 8940, Dell; Round Rock, TX, United States). Stimuli were presented on a 27-inch liquid crystal display (FlexScan, Eizo; Ishikawa, Japan; 1920 × 1,200 pixels; refresh rate 60 Hz). The distance between the monitor and the participants was fixed at approximately 57 cm using a headrest (T.T.K. 930a, Takei Scientific Instrument; Tokyo, Japan). Responses were recorded using a response box (RB-740, Cedrus; San Pedro, CA, United States) that measures RT with a resolution of 2–3 ms.

### Procedures

All participants completed the gaze-cueing task before the emotion-recognition task.

#### Gaze-Cueing Task

The present study used a gaze-cueing paradigm similar to the approach established by Uono et al. (2009a,b); an example trial is shown in Figure 1. First, a fixation cross was presented at the centre of the display for 600 ms. Subsequently, a dynamic emotional (angry, fearful and happy) or neutral face with the eyes gazing to the right or left was presented as a cue stimulus. After 83 ms, a target letter T appeared to the left or right of the cue. The participants were asked to press a button as quickly and accurately as possible when they found the target. The time interval from target appearance to button response was used as the RT. The target and cue remained until the participants responded or 1,000 ms elapsed with no response. The participants were instructed that there is no systematic relationship between cue direction and target location. They were asked to fixate on the centre of the screen as much as possible during each trial. Twelve practice trials were performed to familiarise each participant with the procedure. The gaze-cueing task consisted of six blocks of 48 trials. We included 32 catch trials, in which the target did not appear, to hinder a predictive response to the target appearance. Therefore, 32 trials were performed under each emotional-congruency condition. The trial order was pseudo-randomised and balanced across participants. Participants rested for as long as they wanted between blocks.

#### Emotion-Recognition Task

The emotion-recognition task was similar to the approach used in previous studies (Uono et al., 2017). In total, 48 photographs of faces expressing six basic emotions (anger, disgust, fear, happiness, sadness or surprise) from four Caucasian and four Asian models were selected from two photograph sets (Ekman and Friesen, 1976; Matsumoto and Ekman, 1988). A face photograph was presented at the centre of the display; written labels of the six emotions were presented to its left and right sides. To avoid any confusion regarding the buttons assigned to the six emotion labels, participants were asked to indicate which of the emotion labels best described the emotion expressed in the face photograph; an experimenter carefully recorded their verbal response using six buttons assigned to the emotion labels on a keyboard. Participants were asked to carefully consider all labels for each face photograph. We confirmed that participants understood the meanings of the emotion labels by asking them to illustrate appropriate feelings, context and bodily responses. Two practise trials were performed to familiarise participants with the task requirement. The task did not have a time limit; the photograph and labels remained on the screen until a response was made. No feedback was provided for participants’ responses to prevent them from learning during the task. The participants viewed each photograph once and completed a total of 48 trials. The task consisted of Asian and Caucasian face blocks. The order of the blocks was balanced across participants, with the trial order randomised within each block.

### Data Analysis

#### Gaze-Cueing Effect and Autistic Traits

The mean RT of each correct trial was calculated for each participant. Trials with RT less than 150 ms were excluded (mean ± *SD* = 0.52% ± 0.94) to eliminate the effect of anticipating the target appearance. Furthermore, trials with RT more than three SDs above individual participant mean were excluded from the analyses (1.22% ± 0.90). The error rates were very low in the target (0.41% ± 1.75) and catch (1.50% ± 2.11) trials. We analysed the mean RTs using a repeated-measures analysis of variance with emotion (anger, fear, neutral and happiness) and congruency (congruent and incongruent) as within-participant factors. Additionally, we conducted analysis of covariance with autistic traits (AQ or SRS-2) as a covariate to examine the effect of autistic traits on the gaze-cueing effect. When a significant interaction was found, we conducted follow-up analyses using simple effect tests. Preliminary analysis analysed the effect of age and sex. We have reported the results in a Supplementary Results.

#### Correlations of the Gaze-Cueing Effect With Autistic Traits and Other Participant Characteristics

We planned to test the significance of Pearson’s product–moment correlations between the gaze-cueing effect in each emotion condition and autistic traits (the overall score for AQ and SRS-2), using the scores of TAS-20, LSAS and POMS 2 as covariates; however, we did not find a significant interaction between congruency and emotion and significant associations. Thus, we tested the correlation coefficients between the average of the gaze-cueing effect across emotion conditions and the total scores of each participants’ characteristics. We reported correlation coefficients between the subscale and total scores on each questionnaire and the gaze-cueing effect under each emotion condition (see Supplementary Results).

#### Correlations of the Gaze-Cueing Effect With Emotion-Recognition Ability

The overall accuracy across the six basic emotions and the accuracy in each emotion category were calculated for each participant. Pearson’s product–moment correlations were tested to investigate the associations between the magnitude of the gaze-cueing effect and the ability to recognise emotions under angry and fearful face conditions. We did not test the relationship under the happy face condition because almost all participants answered perfectly for happy faces during the emotion-recognition task. Values of *p* < 0.05 were considered statistically significant unless otherwise specified.

## Results

### Gaze-Cueing Effect and Autistic Traits

The RT results are listed in Table 2. Repeated-measures analysis of variance revealed a significant main effect of congruency, *F*(1,78) = 149.628, *p* < 0.001, *ηp*^2^ = 0.657, indicating that the RTs for the congruent condition were shorter than the RTs for the incongruent condition. There was also a significant main effect of emotion, *F*(3,234) = 43.558, *p* < 0.001, *ηp*^2^ = 0.358. In contrast to our hypothesis, there was no significant interaction between emotion and congruency, *F*(3,234) = 0.268, *p* = 0.848, *ηp*^2^ = 0.003), suggesting that facial expressions do not affect the magnitude of the gaze-cueing effect. Follow-up analysis of the main effect of emotion with Bonferroni correction (*α* = 0.0083) showed that the RTs under emotional face conditions (angry: 309.6 ms; fearful: 309.2 ms; and happy: 306.9 ms) were significantly shorter than the RTs under the neutral face condition (318.1 ms), angry vs. neutral: *t*(158) = 7.226, *p* < 0.001, fearful vs. neutral: *t*(158) = 8.585, p < 0.001, and happy vs. neutral: *t*(158) = 10.140, *p* < 0.001, and that the RTs of the happy face condition were significantly shorter than the RTs of the angry condition, happy vs. angry: *t*(158) = 2.729, *p* = 0.008, happy vs. fearful: *t*(158) = 2.601, *p* = 0.011, and angry vs. fearful: *t*(158) = 0.356, *p* = 0.723. The results suggest that the RTs under the happy face condition were the shortest and those of the angry face condition were the longest. The RTs under the fearful face condition were located in the intermediate position between the happy and angry face conditions. To confirm that gaze cues orient attention under each emotion condition, we conducted *t*-tests with Bonferroni correction for multiple comparisons (*α* = 0.0012). The results revealed that the RTs for the congruent condition were shorter than the RTs for the incongruent condition under all emotion conditions, anger: *t*(78) = 6.977, *p* < 0.001, fear: *t*(78) = 10.100, *p* < 0.001, happiness: *t*(78) = 12.605, *p* < 0.001, and neutral: *t*(78) = 8.895, *p* < 0.001.

**Table 2:** The mean RTs (*SE*) for the gaze-cueing task.

Congruency	Emotion			
	Angry	Fearful	Happy	Neutral
Congruent	302.8 (6.0)	302.1 (6.6)	299.7 (6.3)	310.6 (6.1)
Incongruent	316.4 (7.0)	316.3 (7.0)	314.1 (6.7)	325.7 (7.1)

SE: standard error; RT: reaction time.

When we conducted the analysis of covariance with AQ score as a covariate, there were no significant main effects, *F*(1,77) = 1.281, *p* = 0.261, *ηp*^2^ = 0.016, or interactions involving the AQ score, Emotion*AQ: *F*(3,231) = 2.064, *p* = 0.106, *ηp*^2^ = 0.026, Congruency*AQ: *F*(1,77) = 1.691, *p* = 0.197, *ηp*^2^ = 0.021, and Emotion*Congruency*AQ: *F*(3,231) = 1.367, *p* = 0.254, *ηp*^2^ = 0.017. The interaction between emotion and congruency did not reach significance, *F*(3,77) = 1.504, *p* = 0.214, *ηp*^2^ = 0.019. When the score of SRS-2 was used as a covariate, the results were the same as the results of the AQ score, with no significant main effect, *F*(1,77) = 1.309, *p* = 0.256, *ηp*^2^ = 0.017, or interactions involving the score of SRS-2, Emotion*SRS 2: *F*(3, 231) = 1.037, *p* = 0.377, *ηp*^2^ = 0.013, Congruency*SRS 2: *F*(1, 77) = 1.390, *p* = 0.242, *ηp*^2^ = 0.018, and Emotion*Congruency* SRS 2: *F*(3, 231) = 0.959, *p* = 0.413, *ηp*^2^ = 0.012. The interaction between emotion and congruency was not also significant, *F*(3,77) = 1.211, *p* = 0.306, *ηp*^2^ = 0.015.

### Correlations of the Gaze-Cueing Effect With Autistic Traits and Other Participant Characteristics

**Figure 2:**
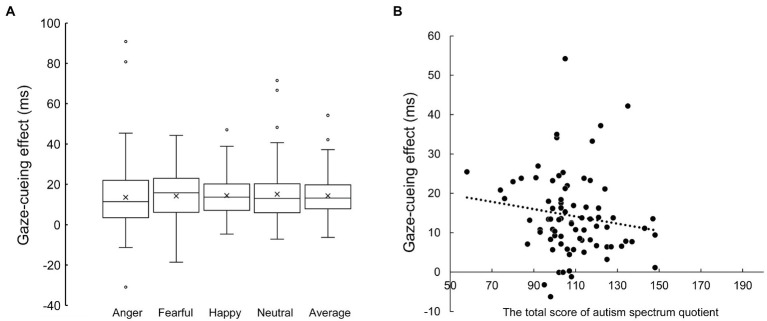
**(A)** The gaze-cueing effect of each emotion condition and their average. **(B)** Scatterplot between the total score of the autism-spectrum quotient (horizontal axis) and the average gaze-cueing effect across the emotional and neutral face condition.

No significant correlations were observed between the gaze-cueing effect across emotion conditions (Figure 2) and the total AQ score (Figure 2; *r* = −0.147, *p* = 0.197), or between the gaze-cueing effect and the SRS-2 for autistic traits (*r* = −0.133, *p* = 0.242). To show that the null hypothesis of the correlation coefficients was highly probable, we report the Bayes factor (BF01), which is the ratio of the marginal likelihood of the null hypothesis to the alternative hypothesis. The Bayes factors were 4.932 and 5.693 for the association between the averaged gaze-cueing effect and the AQ and the SRS-2, respectively. The null hypothesis was about five times more likely than the alternative hypothesis, which is moderate evidence that autistic traits were not associated with the gaze-cueing effect according to Jarosz and Wiley (2014). We also report the correlation coefficients between the subscale scores of the AQ and the gaze-cueing effect under each emotion condition (see Supplementary Results).

The correlation analysis of other participants’ characteristics (social anxiety, alexithymia and mood disturbances) did not detect significant associations with the gaze-cueing effect (LSAS: *r* = −0.208, *p* = 0.066; TAS-20: *r* = −0.145, *p* = 0.201; POMS 2: *r* = −0.006, *p* = 0.955). We also report the correlation coefficients for the relationship between the gaze-cueing effect under each emotion condition and the total scores and the subscale scores for each measurement in Supplementary Tables 1–5.

### Correlations of the Gaze-Cueing Effect With the Emotion-Recognition Ability

The percentage accuracy across the six basic emotions (mean ± *SD* = 72.31% ± 8.38, Table 3) was within the range of previous studies (Uono et al., 2011, 2013, 2017). Correlation analyses did not find a significant association between the magnitude of the gaze-cueing effect and the recognition ability under angry (*r* = 0.064, *p* = 0.577) or fearful (*r* = −0.018, *p* = 0.874) facial expressions.

**Table 3:** The mean percent accuracy (SD) for the emotion-recognition task.

Anger	Disgust	Fear	Happiness	Sadness	Surprise	Average
68.4 (18.7)	41.5 (25.7)	44.9 (27.0)	98.9 (5.0)	83.2 (17.9)	97.0 (7.0)	72.3 (8.4)

SD: standard deviation.

## Discussion

The present study replicated a previous finding that the RTs for the congruent condition are shorter than the RTs for the incongruent condition, even when the gaze direction did not provide a cue to predict the location where the target would appear (see Frischen et al., 2007 for a review). This result suggests that gaze cues reflexively trigger attention orienting in the direction of the eyes. Furthermore, the averaged gaze-cueing effects across emotion conditions in almost all participants (74/79) were above zero (14.30 ms ± 10.39). Although a spatial congruency effect has been found in a localisation task, such that eyes gazing to the right enhances the response for pressing the right button (Downing et al., 2004), this did not contribute to the gaze-cueing effect in the present study because participants were required only to detect the target, not locate it. The stimuli onset asynchrony (SOA) between the last image of the dynamic cues and the target was very short (83 ms). The result and the experimental design in the present study indicate the robustness and reflexiveness of the gaze-cueing effect in adults.

Dynamic emotional (angry, fearful and happy) faces induced a shorter RT than did neutral faces, irrespective of the cue–target congruency. Consistent with this result, previous studies have shown that dynamic facial expressions and gaze cues separately affect the performance in the cueing paradigm (Graham et al., 2010; Chen et al., 2021). Graham et al. (2010) demonstrated that dynamic emotional (disgusted, fearful and happy) faces enhanced the response to the target compared with neutral faces, regardless of whether the target was cued or un-cued, particularly under a condition of low SOA. Chen et al. (2021) showed that dynamic emotional (fearful and happy) faces enhance target detection under both congruent and incongruent conditions compared with neutral faces, even under a high-SOA condition (400 ms). These findings suggest that dynamic emotional faces rapidly alerted the participants and enhanced perceptual processing. Additionally, follow-up analyses showed that the RT was shorter under the happy face condition than under the angry face condition. The RTs under the fearful face condition were located in the intermediate position between the happy and angry face conditions. Previous studies have suggested that happy faces have low-level features that make them visually more salient (e.g., Hess et al., 1997); people have a heightened perceptual sensitivity to happy faces compared to other emotional faces (Maher et al., 2014). Furthermore, a study that investigated the effect of emotional faces with an averted gaze under the anti-saccade paradigm demonstrated that angry faces hold participants’ attention longer compared to neutral faces (Bonifacci et al., 2008). The result in the present study suggests that dynamic emotional faces alert the participants and enhance perceptual processing. While the rapid processing of happy faces might improve participants to respond rapidly to the subsequently presented target, the difficulty in disengaging from threatening faces might delay the response.

The present results did not replicate the influence of emotional facial expressions on the gaze-cueing effect, despite using dynamic facial expressions (angry, fearful and happy) that changed with gaze direction as cues; such expressions have been used in previous studies to demonstrate the enhanced gaze-cueing effect of emotional faces (e.g., Uono et al., 2009a). The ability to recognise the emotion was not associated with the magnitude of the gaze-cueing effect under either angry or fearful face conditions. This finding also suggests that gaze cues trigger reflexive attention orienting. A recent study in a sample of 100 women reported that emotional facial expressions (i.e., angry and fearful faces) did not modulate gaze-triggered attention orienting (Talipski et al., 2020). However, many studies have demonstrated that dynamic presentations of fearful (Tipples, 2006; Uono et al., 2009a; Lassalle and Itier, 2013, 2015b; Neath et al., 2013; McCrackin and Itier, 2018) and other emotional faces (angry: Lassalle and Itier, 2013, 2015b; Liu et al., 2019; Neath et al., 2013; surprised: Lassalle and Itier, 2013, 2015b; and happy: McCrackin and Itier, 2019) enhance attention orienting, compared with neutral faces. While studies involving static faces with emotional expressions have reported mixed findings (see Dalmaso et al., 2020 for a review), McCrackin and Itier (2019) combined the data across experiments; they demonstrated that dynamic negative, as well as positive, facial expressions enhanced the gaze-cueing effect. Thus, we suggest that emotional facial expressions could modulate the magnitude of the gaze-cueing effect as shown in previous studies; several experimental factors may inhibit the integration of emotional facial expression and gaze direction in the present study, as we shall now discuss.

The experimental paradigm in the present study might include several factors that inhibit the enhancement of the gaze-cueing effect by dynamic emotional expressions, although a similar paradigm has been used to demonstrate such an effect (Uono et al., 2009a,b). First, there is a possibility that the use of many emotional expressions disambiguated their emotional impact because our study utilised four types of facial expressions (angry, fearful, happy and neutral) in the same block. A previous study demonstrated that static fearful faces enhance the gaze-cueing effect under the condition that they did not appear frequently compared to neutral faces (Kuhn et al., 2016). Second, the present study used the experimental setting, in which facial expression changed from neutral to emotional simultaneously with increasing gaze deviation. Lassalle and Itier (2015b) showed that the gaze-cueing effect was enhanced by emotional facial expressions when such expressions changed after gaze deviation; however, the effect was not enhanced when the expressions changed concomitantly with or before gaze deviation. McCrackin and Itier (2019) also demonstrated enhancement of the gaze-cueing effect using emotional faces under the cue sequence that facial expressions changed after a deviated gaze, while Talipski et al. (2020) did not find emotional enhancement of the gaze-cueing effect under the cue sequence that gaze direction changed after emotional faces were presented. Thus, the cue sequence that facial expressions changed after the gaze deviation could be more ecologically valid to induce emotional enhancement of the gaze-cueing effect because people attend to an object and then express emotion depending on the emotional value of the object. Third, the SOA (between 20% emotional faces with an averted gaze and target appearance) used in the present study was small (205 ms). Graham et al. (2010) demonstrated the enhancement of the gaze-cueing effect by dynamic emotional expressions for high SOA but not for low-SOA conditions (<275 ms). This finding suggests that the time interval between deviating the gaze and appearance of the target is an important factor determining whether emotional faces modulate the gaze-cueing effect. Fourth, a characteristic of the task demand, where participants detected the target (rather than localising it), might have led to the null finding. A recent study showed that emotional facial expressions modulate the gaze-cueing effect in a localisation task but not in a discrimination task (Chen et al., 2021). Considering that gaze direction embedded in emotional faces indicates where an important object is but not whether an object is present or absent, the localisation task might be more ecologically valid to detect the effect of emotional faces. A future study should identify which factors determine the magnitude of the gaze-cueing effect of emotional faces.

The degree of autistic traits overall did not affect the gaze-cueing effect for neutral or emotional faces. No significant correlations were detected between autistic traits and the gaze-cueing effect. Consistent with this result, a recent study did not reveal any emotional enhancement of the gaze-cueing effect or any associations between the gaze-cueing effect of emotional faces and autistic traits when gaze direction changed after emotional faces were presented (Talipski et al., 2020). Studies using neutral face cues also did not find any association of autistic traits with the gaze-cueing effect (Lachat et al., 2012a; de Araujo et al., 2021; however, see Bayliss et al., 2005). McCrackin and Itier (2019) reported that when facial expression changed after gaze deviation, positive and negative emotional faces enhanced the gaze-cueing effect and a higher score for attention to detail is associated with a smaller gaze-cueing effect of happy faces. Those researchers suggested that reduced holistic perception prevents the processing of emotional faces in people with high-autistic traits. The lack of significant relationships between autistic traits and the gaze-cueing effect of emotional faces in the present study might be explained by the lack of enhancement of the gaze-cueing effect by emotional faces (we note that the score of attention to detail was associated with the gaze-cueing effect of neutral rather than emotional faces, although this finding in a large number of exploratory analyses should be interpreted with caution; see Supplementary Results). In addition, some studies have demonstrated that the degree of autistic traits modulates the magnitude of the gaze-cueing effect for specific types of targets. For example, Bayliss and Tipper (2005) found that participants in a low-AQ group showed a larger gaze-cueing effect in response to an integrated target stimulus (faces and tools) compared with a scrambled target stimulus, while participants in the high-AQ group showed the opposite pattern. Zhao et al. (2015) utilised auditory targets (voice and tone); they demonstrated that an enhancement of the gaze-cueing effect under voice versus tone target conditions disappeared earlier in the high-AQ group than in the low-AQ group. These studies suggest that the magnitude of the gaze-cueing effect is modulated by the degree of participants’ autistic traits through their interests in the target objects. The use of a more ecologically valid paradigm might be needed to reveal the association between autistic traits and the gaze-cueing effect.

The present study investigated the effect of participant characteristics (the degree of social anxiety, alexithymia and emotional disturbance) in addition to autistic traits. The correlation coefficients revealed no significant influences of these variables on the magnitude of the gaze-cueing effect. The findings of the present study suggest that the effect of individual differences in emotional aspects on the gaze-cueing effect is not robust. Consistent with this proposition, recent studies with large sample sizes have reported null findings for the effects of social anxiety, trait anxiety and depression (McCrackin and Itier, 2019; Talipski et al., 2020). Early studies with small sample sizes reported contradictory findings. Uono et al. (2009a) demonstrated that trait and state anxiety do not modulate the gaze-cueing effect of dynamic fearful faces, while some pioneering studies have shown an enhanced the gaze-cueing effect in people who have high anxiety (Putman et al., 2006; Tipples, 2006). The gaze-cueing effect measured in a controlled experiment may not be a robust and elaborate representation of current social cognitive function. However, the effect of individual differences in emotional aspects of human cognition on the gaze-cueing effect might be affected by several experimental variables, as discussed above. The influence of individual differences in emotional traits should be explored further, considering that the present study could not replicate the enhancement of the gaze-cueing effect by dynamic facial expressions.

There are several points to be noted. First, we employed a dimensional approach to investigate the relationship between autistic traits and the gaze-cueing effect. Thus, the present study included only a small number of participants with autistic traits above the clinical cut-off. However, previous studies using the same approach have reported a significant relationship between autistic traits and the gaze-cueing effect (e.g., McCrackin and Itier, 2019). Second, the cue stimuli were created from only two people (one man and one woman). Although we chose the cue stimuli because they demonstrated enhancement of the gaze-cueing effect under the fearful face condition in previous studies (Uono et al., 2009a,b), including the cue stimuli from many people is ideal for reproducibility and generalisability. Third, the sex of the participants was biased towards women (58 women and 21 men) and the age range was large (18–55 years). Although these factors did not affect emotional enhancement of the gaze-cueing effect (see Supplementary Results), further studies should consider more appropriate sampling for generalisability. Finally, as discussed above, several experimental factors might have undermined the difference in the gaze-cueing effect between neutral and emotional faces. Future studies should consider using more ecologically valid dynamic cue sequence (i.e., emotional expression after gaze aversion; McCrackin and Itier, 2019) and task (i.e., localisation; Chen et al., 2021) and including a high-SOA condition (e.g., approximately 500 ms; Graham et al., 2010).

In conclusion, the present study showed the robustness and reflexiveness of the gaze-cueing effect in adults. However, emotional expressions (anger, fear, and happiness) did not enhance the gaze-cueing effect in this study. Additionally, individual differences in autistic traits, and emotional characteristics (social anxiety, alexithymia, and emotional disturbances) did not influence the magnitude of the gaze-cueing effect. Although the ability to orient attention in the direction of another’s gaze is a fundamental function of social development, the gaze-cueing effect measured in a controlled experiment might not be an elaborate representation of the current social cognitive function, at least in typically developing adults.

## Data Availability Statement

The raw data supporting the conclusions of this article will be made available by the authors, without undue reservation.

## Ethics Statement

The studies involving human participants were reviewed and approved by the Ethics Committee of National Center of Neurology and Psychiatry, Japan. The patients/participants provided their written informed consent to participate in this study.

## Author Contributions

SU, YE, SH, MU, and TO conceived and designed the experiments. SU, YE, SH, MT, and TO performed the experiments. SU analysed the data and wrote the first draft of the manuscript. All authors contributed to the article and approved the submitted version.

## Funding

This work was supported by JSPS KAKENHI (20K03478, 20K07917, 20K14058, and 21K13760), Japan Health Research Promotion Bureau Research Fund for Young Investigators (JH2021-Y-10), and Intramural Research Grant for Neurological and Psychiatric Disorders of NCNP (2-7) and Meiji Yasuda Mental Health Foundation.

## Conflict of Interest

The authors declare that the research was conducted in the absence of any commercial or financial relationships that could be construed as a potential conflict of interest.

## Publisher’s Note

All claims expressed in this article are solely those of the authors and do not necessarily represent those of their affiliated organizations, or those of the publisher, the editors and the reviewers. Any product that may be evaluated in this article, or claim that may be made by its manufacturer, is not guaranteed or endorsed by the publisher.
