# Supplementary material for: No Influence of Emotional Faces or Autistic Traits on Gaze-Cueing in General Population
Source: Front Psychol. 2022 Apr 26;13:864116. doi: 10.3389/fpsyg.2022.864116 (PMC9088812; doi:10.3389/fpsyg.2022.864116)
Supplement: Supplementary file 1 [file Table_1.DOCX]

**Supplementary results for the gaze-cueing task**

We analyzed the effect of age and sex on the gaze-cueing effect. Although age and sex influenced on the gaze-cueing effect and the RTs respectively, the emotional enhancement of the gaze-cueing effect was not affected by these factors.

*Age*

The ANCOVA with congruency and emotion as within-participants factors and age as a covariate was conducted. As the analysis reported in the main text, there was significant main effect of emotion, *F*(3, 231) = 5.162, *p* = .002 , *ηp^2^* = .063. There was also a significant d main effect of age, *F* (1, 77) = 4.965, *p* = .029, *ηp^2^* = .061, and interaction between congruency and age, *F* (1,77) = 11.754, *p* < .001, *ηp^2^* = .132. There were no other significant effects. As follow-up analysis of the interaction, we investigated correlations between age and the RTs under each congruency condition and the averaged gaze-cueing effect. Chronological age was positively associated with the RTs under congruent (*r* = .224, *p* = .047) and incongruent conditions (*r* = .265, *p* = .018). The positive correlation between the averaged gaze-cueing effect across emotion condition and age was also found (*r* = 364, *p* < .001). These results suggested that the older people showed the slower RTs in general and the RT difference between congruent and incongruent conditions increase with ageing.

To investigate the possibility that older but not younger participants showed the gaze-cueing effect, we further conducted the ANOVA with congruency and emotion as within-participants factors in younger participants (< 30 years old). This analysis replicated the results reported in whole participants. There was a significant main effect of congruency, *F*(1, 59) = 99.829, *p* < .001, *ηp^2^* = .629, indicating that the RTs for the congruent condition were shorter than the RTs for the incongruent condition. There was also a significant main effect of emotion, *F*(3, 177) = 37.561, *p* < .001, *ηp^2^* = .389. In contrast to our hypothesis, there was no significant interaction between emotion and congruency, *F*(3, 177) = .398, *p* = .755 , *ηp^2^* = .007, suggesting that facial expressions do not affect the magnitude of the gaze-cueing effect.

*Sex*

The ANOVA with congruency and emotion as within-participants factors and sex as a between-participant factor was conducted. There was a significant main effect of congruency, *F*(1, 77) = 111.197, *p* < .001, *ηp^2^* = .591, indicating that the RTs for the congruent condition were shorter than the RTs for the incongruent condition. There was also a significant main effect of emotion, *F*(3, 231) = 28.160, *p* < .001, *ηp^2^* = .268. In contrast to our hypothesis, there was no significant interaction between emotion and congruency, *F*(3, 231) = .084, *p* = .969, *ηp^2^* = .001, suggesting that facial expressions do not affect the magnitude of the gaze-cueing effect. We found significant interaction between sex and emotion, *F*(3, 231) = 7.196, *p* < .001, *ηp^2^* = .085, though the main effect of sex was not significant, *F*(3, 231) = .809, *p* = .371, *ηp^2^* = .010. The follow-up analysis of this interaction with Bonferroni correction (α = .0083) revealed that the RTs of emotional faces were shorter than those of neutral faces (*ps* < .001) in women. For men, the RTs of happy faces were shorter than those of neutral and anger faces (*ps* < .001). The results suggested that sex have differential influences on the processing of emotional facial expressions, though sex had no influence of the effects involving congruency, congruency*sex: *F*(1, 77) = .195, *p* = .660, *ηp^2^* = .003, congruency*gaze*sex: *F*(3, 231) = .441, *p* = .724, *ηp^2^* = .006. Further studies are needed to conclude the effect of sex because the participants in the present study were biased to women (21 men and 58 women).

**Supplementary results for correlation analyses**

The association between the subscale scores of the autism spectrum quotient (Table. 1), Liebowitz social anxiety scale (Table. 2), Toronto alexithymia scale (Table. 3), profile of mood states-second edition (Table. 4), and Wechsler adult intelligence scale-fourth edition (Table. 5) and the gaze-cueing effect.

| **Supplementary Table 1.** | | | | | | |
| --- | --- | --- | --- | --- | --- | --- |
| The correlational coefficients between the gaze-cueing effect and the scores of the AQ | | | | | | |
|  | AQ | | | | | |
| Emotion | Social skill | Attention switching | Attention to detail | Communication | Imagination | Total |
| Angry | .026 | .049 | .006 | -.072 | -.020 | -.004 |
| Fearful | .012 | -.175 | -.217 | -.134 | -.033 | -.146 |
| Happy | -.043 | -.126 | .049 | -.122 | -.053 | -.082 |
| Neutral | -.092 | -.185 | -.269 | -.115 | -.157 | -.224 |
| Average | -.029 | -.130 | -.148 | -.142 | -.088 | -.147 |
| AQ: autism spectrum quotient | | | | | | |

| **Supplementary Table 2.** | | | |
| --- | --- | --- | --- |
| The correlational coefficients between the gaze-cueing effect and the scores of the LSAS | | | |
|  | Social anxiety | | |
| Emotion | Fear | Avoidance | Total |
| Angry | -.087 | -.109 | -.105 |
| Fearful | -.234 | -.258 | -.264 |
| Happy | -.136 | -.129 | -.142 |
| Neutral | -.110 | -.150 | -.139 |
| Average | -.179 | -.208 | -.208 |
| LSAS: Liebowitz social anxiety scale | | | |

| **Supplementary Table 3.** | | | | |
| --- | --- | --- | --- | --- |
| The correlational coefficients between the gaze-cueing effect and the scores of the TAS-20 | | | | |
|  | Alexithymia | | | |
| Emotion | DIF | DDF | EOT | Total |
| Angry | -.068 | -.030 | -.209 | -.131 |
| Fearful | -.005 | .148 | -.166 | .003 |
| Happy | -.240 | -.104 | .064 | -.210 |
| Neutral | -.132 | .057 | -.182 | -.113 |
| Average | -.136 | -.027 | -.218 | -.145 |
| DIF: difficulties identifying feelings; DDF: difficulties describing feelings; EOT: externally oriented thinking; the TAS-20: Toronto alexithymia scale | | | | |

| **Supplementary Table 4.** | | | | | | | | |
| --- | --- | --- | --- | --- | --- | --- | --- | --- |
| The correlational coefficients between the gaze-cueing effect and the sub-scale scores of the POMS-2 | | | | | | | | |
|  | Mood disturbances | | | | | | |  |
| Emotion | A-H | C-B | D-D | F-I | T-A | V-A | F | TDM |
| Angry | .028 | .054 | -.037 | -.081 | -.031 | .102 | .045 | -.041 |
| Fearful | .069 | .135 | .034 | .048 | -.029 | -.078 | .079 | .077 |
| Happy | -.096 | -.088 | -.117 | -.176 | -.032 | .039 | -.051 | -.118 |
| Neutral | -.045 | .029 | .000 | -.058 | .066 | -.153 | -.003 | .045 |
| Average | -.007 | .052 | -.034 | -.084 | -.006 | -.027 | .029 | -.006 |
| A-H: anger-hostility; C-B: confusion-bewilderment; D-D: depression-dejection; F: friendliness; F-A: fatigue-inertia, POMS-2: profile of mood states, second edition; T-A: tension-anxiety; TDM: total mood disturbance; V-A: vigor-activity (VA) | | | | | | | | |

| **Supplementary Table 5.** | | | | | |  |
| --- | --- | --- | --- | --- | --- | --- |
| The correlational coefficients between the gaze-cueing effect and the scores of the WAIS-IV | | | | | |  |
|  | Intellectual ability | | | | |  |
| Emotion | VCI | PRI | WMI | PSI | FIQ |  |
| Angry | .004 | -.044 | -.100 | -.154 | -.089 |  |
| Fearful | .003 | .013 | -.134 | -.010 | -.019 |  |
| Happy | -.153 | -.021 | -.069 | -.173 | -.143 |  |
| Neutral | -.004 | -.070 | -.219 | -.105 | -.128 |  |
| Average | -.036 | -.045 | -.178 | -.147 | -.124 |  |
| FIQ: full-scale intelligence quotient; PRI: perceptual reasoning index; PSI: processing speed index; VCI: verbal comprehension index; WAIS-IV: Wechsler adult intelligence scale, fourth edition; WMI: working memory index | | | | | |  |
|  |  |  |  |  |  |  |
